# A Nutrient-Dense Diet with Optimal Protein Level Restores Goblet Cells and Alleviates Microbial Dysbiosis in a Mouse Model of Colitis

**DOI:** 10.1016/j.tjnut.2026.101546

**Published:** 2026-04-22

**Authors:** Lingfan Jiang, Jiaxuan Li, Abrory AC Pramana, Yuan-Xiang Pan, Wenyan Mei, Hong Chen

**Affiliations:** 1 Department of Food Science and Human Nutrition, University of Illinois Urbana-Champaign, Urbana, IL, United States; 2 Division of Nutritional Sciences, University of Illinois Urbana-Champaign, Urbana, IL, United States; 3 Department of Biochemistry, Faculty of Medicine, Public Health, and Nursing, Universitas Gadjah Mada, Yogyakarta, Indonesia; 4 Department of Comparative Biosciences, College of Veterinary Medicine, University of Illinois Urbana-Champaign, Urbana, IL, United States

**Keywords:** protein diet, goblet cell, colon inflammation, gut microbiota, Dubosiella

## Abstract

**Background:**

Chronic intestinal inflammation is a key driver of colitis-associated colorectal cancer. A mouse model with intestinal epithelial cell–specific knockout (KO) of heterogeneous nuclear ribonucleoprotein I (*hnRNPI* KO) spontaneously develops colitis and reduces goblet cell numbers, mimicking the pathological symptoms observed in humans with chronic intestinal inflammation, making the *hnRNPI* KO mouse an invaluable model of gut inflammation. Although higher dietary protein has been associated with lower inflammation and restoration of immune adaptation, the mechanisms remain unclear.

**Objectives:**

This study aimed to investigate the effects of optimized dietary protein on colonic goblet cell functions and on correcting gut dysbiosis in the *hnRNPI* KO mouse model.

**Methods:**

Male wild type (WT) and *hnRNPI* KO mice were fed either a control protein diet (CON; 14.41% kcal protein) or a nutrient-dense modified diet (MOD; 28.83% kcal protein) for 22 wk. Body weight, food intake, and colon histology were assessed. Goblet cells and mucin content were quantified using Alcian Blue/periodic acid–Schiff staining. CD4^+^ T-cell subpopulations were measured by flow cytometry. Gut microbial composition was identified using 16S rRNA amplicon sequencing and analyzed using QIIME2 and phyloseq. Two-way analysis of variance with Tukey’s multiple comparisons was used to assess diet and genotype interactions.

**Results:**

Feeding the MOD restored goblet cell numbers and mucin content in KO mice (*P* < 0.05). MOD-fed KO mice had significantly lower IL-17^+^ interferon-γ^+^ CD4^+^ T-cell subset in the colon than the WT-MOD mice (873 and 1885 cells, respectively, *P* < 0.05). Feeding KO mice MOD significantly increased fecal *Dubosiella* sp. (24% in MOD-KO; 12% in CON-KO; *P* < 0.05) and decreased *Lachnospiraceae NK4A136* abundance (0.18% in MOD-KO; 2.3% in CON-KO; *P* < 0.05).

**Conclusions:**

An optimized dietary protein restores colon goblet cells, modulates immune responses, and alleviates gut microbial dysbiosis. Our results highlight the benefits of optimized dietary protein in ameliorating intestinal inflammation and potentially enhancing gut health.

## Introduction

Colorectal cancer (CRC) is the third most commonly diagnosed cancer worldwide and a leading cause of cancer-related death. One of the most significant risk factors for CRC is chronic inflammation of the intestinal mucosa, a hallmark of inflammatory bowel diseases (IBD) such as ulcerative colitis and Crohn’s disease [1]. Chronic colitis leads to continuous epithelial damage and regeneration, which increases the likelihood of dysplasia and neoplastic transformation [2,3].

In the healthy intestine, intestinal epithelial cells (IECs) play a central role in regulating barrier function, coordinating mucosal immunity, and interacting with the intestinal microbiota [4,5]. Recent studies have identified heterogeneous nuclear ribonucleoprotein I (hnRNPI), also known as polypyrimidine tract binding protein 1, as a key RNA-binding protein responsible for posttranscriptional regulation, mRNA splicing, and immune adaptation [6,7]. Knockout (KO) of the *hnRNPI* gene in IECs leads to spontaneous early-onset colitis in mice, characterized by increased crypt cell proliferation, immune cell infiltration, and reduced number of goblet cells [8]. The phenotype of the *hnRNPI* KO mouse resembles human IBD in many aspects, with chronic colonic inflammation, goblet cell loss, epithelial barrier dysfunction, immune dysregulation, gut microbiota dysbiosis, and increased risk of CRC [8,9]. Mechanistically, *hnRNPI* regulates the expression of IL-1 receptor-associated kinase 1 (IRAK1), a key component of the toll-like receptor/nuclear factor κB signaling axis. In *hnRNPI* KO mice, upregulation of IRAK1 leads to dysregulated cytokine signaling and failure of neonatal immune adaptation, which, in turn, triggers inflammation [10].

Goblet cells are responsible for secreting mucins and forming a protective mucus layer on the intestinal epithelium. Goblet cells not only act as a physical barrier but also participate in immune regulation by delivering luminal antigens to immune cells in a tolerogenic manner [11]. Disruption of goblet cell function or mucin production increases intestinal permeability and enhances exposure to microbial antigens, thereby exacerbating immune responses [12,13]. Inflammation-induced goblet cell depletion has been associated with poor clinical prognosis and increased susceptibility to CRC in patients with IBD [14,15].

Spatial profiling has consistently shown the heterogeneous immune landscape in the inflamed colon. During colitis, the mucosa is infiltrated by distinct CD4^+^ T-cell subsets, including proinflammatory helper T-cell–1 (Th1) and Th17 cells, as well as regulatory T cells (Treg), which work to suppress excessive immune responses [16,17]. The Treg/Th17 balance is particularly critical: disruption of this balance drives disease progression, whereas restoration of Treg dominance promotes tissue healing [3]. This axis is now considered a target for both pharmacologic and nutritional interventions in IBD. Despite these insights, the underlying immunological changes, particularly in atypical CD4^+^ T-cell subsets such as IL17^-^ interferon-γ^-^ (IFNγ^-^) cells, remain understudied.

Gut microbiota is also a contributor to colitis pathogenesis. Dysbiosis, defined as an imbalance in microbial composition, leads to an increase in pathogenic microorganisms and a reduction in beneficial microorganisms, accompanied by promoting inflammation and compromising immune tolerance [18,19]. Microbial fermentation products, such as short-chain fatty acids, tryptophan metabolites, and amino acid derivatives, interact with the host and modulate mucosal immunity [20]. A recent study has found that gut microbiota dysbiosis in *hnRNPI* KO mice challenged with dextran sulfate sodium (DSS), shown by lower abundance in *Dubosiella* sp., increased the CRC risk [9].

Diet can affect intestinal physiology, modulate immunity, and influence gut microbiota. Protein, as an essential macronutrient, influences gut health through multiple mechanisms. A diet with moderately enhanced protein level (30% of energy) has been shown to benefit epithelial repair and to reduce inflammation [21]. On the other hand, having an excessive protein intake (>35% kcal) can worsen colitis by generating toxic nitrogenous metabolites and reducing microbial diversity [[21], [22], [23], [24]]. A recent study showed that a nutrient-dense, moderate-protein diet can suppress inflammatory cytokines in *hnRNPI* KO mice [10]. However, the interplay between dietary protein and microbial species, including their metabolites, is only beginning to be understood.

In this study, we employed an IEC-specific *hnRNPI* KO mouse model to examine the effects of a modified nutrient-enriched diet (MOD, 28.83% kcal protein) on chronic colitis, including whether it could reverse goblet cell loss, restore mucin content, rebalance T-cell subsets, and alter microbial composition in the context of chronic colitis. We specifically assessed changes in epithelial barrier function, proinflammatory signaling, regulatory CD4^+^ T-cell populations, and microbial taxa. Our study aims to elucidate the immunometabolism mechanisms by which protein nutrition modulates inflammation and inform dietary strategies for the management of IBD and the reduction of CRC risk.

## Methods

### Animal and diet

All animal protocols were approved by the Institutional Animal Care and Use Committee at the University of Illinois Urbana-Champaign (Protocol #20119). Mice harboring an IEC-specific *hnRNPI* deletion (*hnRNPI*^flox/flox^; ^VillinCre/+^, henceforth KO) were generated by crossing male *hnRNPI*^flox/flox^; *Villin*^Cre/+^ mice with female *hnRNPI*^flox/flox^ mice, as previously described [8]. Genotypes were confirmed by PCR using primer sets specific to the floxed *hnRNPI* allele and the *Villin*^Cre/+^ allele.

Pups were weaned and divided into different cages on the basis of genotype and sex at 21 d of age. After weaning, each mouse was ear-tagged with a unique identification number. Mice were separated by sex and genotype and housed in polycarbonate cages with aspen bedding in a temperature- and humidity-controlled room in Animal Care Facilities at the University of Illinois at Urbana-Champaign. All mice were fed a purified American Institute of Nutrition-93 (AIN-93) diet (D10012M, Research Diet Inc.) and water ad libitum in a 12-h light environment during breeding.

For dietary treatments, 2 diets were designed and purchased from Research Diets, Inc:

1. Control protein diet (CON): AIN-93M diet provides 14.41% kcal from protein, 75.26% kcal from carbohydrate, and 10.32% from fat (Table 1).

**TABLE 1:** Composition of experimental diets

Ingredients	CON	MOD
Casein (g/kg)	140.00	280.00
Soybean oil (g/kg)	40.00	80.00
Maltose dextrin (g/kg)	230.00	141.44
Corn starch (g/kg)	151.00	48.50
Dextrose (g/kg)	240.00	200.00
Sucrose (g/kg)	100.00	80.00
Cellulose (g/kg)	50.00	72.00
Salt mix, AIN-93MMX (g/kg)	35.00	70.00
Vit. mix, AIN-93 (g/kg)	10.00	20.00
L-cystine (g/kg)	1.50	3.00
Choline bitartrate (g/kg)	2.50	5.00
FD&C dye (g/kg)	—	0.06
Total (g/kg)	1000.00	1000.00
Protein (% kcal)	14.41	28.83
Fat (% kcal)	10.32	20.64
Carbohydrate (% kcal)	75.26	50.52
Total energy (kcal/kg)	3850	3850

Abbreviations: CON, control protein diet (14.41% kcal from protein); MOD, nutrient-dense modified diet (28.83% kcal from protein); AIN-93MMX: AIN-93 Mineral Mix; FD&C: Food, Drug, and Cosmetic Act. [10].
2. Nutrient-dense modified diet (MOD): a customized diet providing 28.83% kcal from protein, 50.52% kcal from carbohydrate, and 20.64% kcal from fat (Table 1).

The composition of the treatment diet was determined in reference to previously published studies; specifically, a protein content of 28.83% kcal was selected for its demonstrated benefits on epithelial repair, and reduction of colon inflammation and permeability [21,25]. To ensure sufficient vitamins and minerals needed for maintaining nutrient balance and to properly use the increased amounts of macronutrients, salt mix and vitamin mix were also increased [10].

Dietary treatments began at ∼5 wk of age and ended at around 27 wk of age. Only male mice were used in this study. Both wild type (WT) and KO mice were randomly assigned to the control diet (CON) and the MOD as previously published [10], resulting in 4 treatment groups: WT-CON (*n* = 11), WT MOD (*n* = 11), KO CON (*n* = 11), and KO-MOD (*n* = 10) with 3–4 mice per cage.

Mice remained on their assigned diets for ∼22 wk. The body weight of each mouse was measured weekly throughout the experimental period. Food intake was assessed weekly per cage by weighing the remaining food at the end of each week and comparing it with the initial food weight provided at the beginning of the week. Weekly food consumption was measured for each cage; difference between the initial and remaining food weights was calculated by dividing the food consumed by the number of mice in that cage. At the study end, mice were killed by CO_2_ asphyxiation, and the entire colon was collected for further analyses.

### Colon tissue sampling

The colon, excised from the ileocecal junction to the anus, was flushed with phosphate-buffered saline (PBS). A distal colon segment (∼1/8 total length) was collected, opened longitudinally, flattened, flash-frozen in liquid nitrogen, and stored at –80°C. Another 1 of 8 segment was embedded in optimal cutting temperature (OCT) compound for histology. The remaining colon was used to isolate immune cells.

### Histological analysis

Distal colon samples were embedded in OCT and then sectioned into 10-μm–thick slices using a Leica CM3050 S Cryostat. Tissue sections were stained with hematoxylin and eosin (H&E) following a previously published procedure [10]. Sections were deparaffinized and rehydrated as follows: xylene was changed 3 times for 5 min each, followed by 100% alcohol for 3 times for 2 min each, 95% alcohol for 2 min, and 70% alcohol for 2 min. Further, sections were stained by H&E (ThermoFisher Scientific). Slides were scanned using a Nanozoomer Digital Pathology system (Hamamatsu Photonics, K.K.).

### Alcian Blue/periodic acid–Schiff staining

Sectioned tissue was deparaffinized and rehydrated using the same procedure as in the histological analysis, then stained with Alcian Blue/periodic acid–Schiff (AB/PAS) according to the modified protocol. Tissue sections were incubated in 3% acetic acid for 3 min, then in 1% Alcian Blue at pH 2.5 for 10 min. Sections were then rinsed with tap water and incubated in 0.5% periodic acid solution for 5 min. Then, sections were rinsed with tap water and incubated in Schiff reagent McMannus solution for 5 min. Then, tissue sections were rinsed with warm tap water and stained with Hematoxylin solution. Slides were scanned using a Nanozoomer Digital Pathology system (Hamamatsu Photonics, K.K.) with 20× and 40× magnification. Goblet cells were identified by purple staining. The number of goblet cells was quantified by counting them in 5 crypts per section, across 7 sections per animal. Goblet cells and mucin content were quantified after scanning.

### Cell isolation and purification

Whole colons were flushed with PBS, opened longitudinally, and cut into 2–3 cm segments. Segments were stirred at 37°C for 15 min in the extraction solution [Roswell Park Memorial Institute (RPMI) medium, fetal bovine serum (FBS), EDTA, and dithiothreitol] to release the epithelial cells from the lamina propria, which were collected by centrifugation. The remaining tissue was minced and digested in RPMI with FBS, dispase II, and collagenase II. Cells were filtered, pelleted, and purified by density gradient centrifugation with 40% and 80% Percoll solutions. The immune cell layer formed at the interphase was collected for flow cytometry.

### Flow cytometry

Cells were blocked with antimouse CD16/32 and stained for the following surface markers:phycoerythrin (PE) antimouse CD45 and allophycocyanin (APC) antimouse CD4. Intracellular staining was performed using Foxp3 Fixation/Permeabilization Buffer (Thermo Fisher) with PE/Cy7 antimouse IFN-γ, PE antimouse IL-17A,fluorescein isothiocyanate (FITC) antimouse Foxp3, and APC anti-Helios. Zombie Aqua was used to exclude dead cells. Flow cytometry data were acquired using an Attune NxT Acoustic Focusing Cytometer and analyzed with FlowJo v10.

### 16S rRNA gene sequencing

For DNA extraction, fecal pellets were collected and flash-frozen in liquid nitrogen. DNA was extracted using the Quick-DNA Fecal/Soil Microbe Microprep Kit (D6012, Zymo Research) following the manufacturer’s instructions. The extracted DNA was quantified using a NanoDrop 2000 Spectrophotometer (Thermo Fisher Scientific). As outlined in previous studies, sequencing was conducted at the W. M. Keck Center for Comparative and Functional Genomics, part of the University of Illinois Biotechnology Center. The hypervariable V3–V4 regions of the 16S rRNA gene (357F/805R: 5'-CCTACGGGNGGCWGCAG, 5'-GACTACHVGGGTATCTAATCC) were amplified using a Fluidigm Access Array (Fluidigm). After amplification, DNA amplicons from all samples were pooled in equimolar concentrations. The pooled amplicons underwent size selection on a 2% agarose E-gel (Life Technologies) and were subsequently purified with a Qiagen gel purification kit (Qiagen). After purification and normalization, the amplicon pools were assessed using an Agilent Bioanalyzer (Advanced Analytics). Final sequencing was carried out using an Illumina MiSeq Nano V2 platform with 2 × 250 bp paired-end reads (Illumina Inc), following established sequencing protocols. Sequencing data from the sequencing process were demultiplexed using bcl2fastq v2.20 conversion software (Illumina Inc).

### Sequencing data analysis

Demultiplexed sequences were analyzed using QIIME2 (v.2024.2) and phyloseq (v. 1.52.0). Chao1 and Shannon indices were used to assess alpha diversity, and between-sample beta diversity was visualized using nonmetric multidimensional scaling on the basis of Bray–Curtis dissimilarities and Binary Jaccard dissimilarities. To assess differences in alpha diversity among groups, pairwise Wilcoxon rank sum tests were performed, and the resulting *P* values were corrected for multiple comparisons using the Benjamini–Hochberg (BH) false discovery rate (FDR) method. Pairwise differences in beta diversity between groups were assessed using permutational multivariate analysis of variance (ANOVA) based on Bray–Curtis distances with 999 permutations, and *P* values were likewise corrected using the BH FDR method. Differentially abundant taxa were identified using linear discriminant analysis (LDA) effect size (LEfSe), with significance set at *P* values < 0.05 and a logarithmic LDA score cutoff of 2.0. The LDA score estimates the effect size of each differentially abundant feature in each taxon and identifies microbial taxa signatures in each group.

### Statistical analysis

A 2-way repeated-measures ANOVA was followed by Tukey’s multiple comparisons test to evaluate the effects of diet and genotype over time. For food intake, colon parameters, histology, and flow cytometry results, 2-way ANOVA was performed with genotype and diet as fixed factors, followed by Tukey’s multiple comparisons when interaction effects were significant. *P* < 0.05 was considered statistically significant.

## Results

### Dietary protein modification attenuates genotype-dependent differences in body weight

**FIGURE 1:**
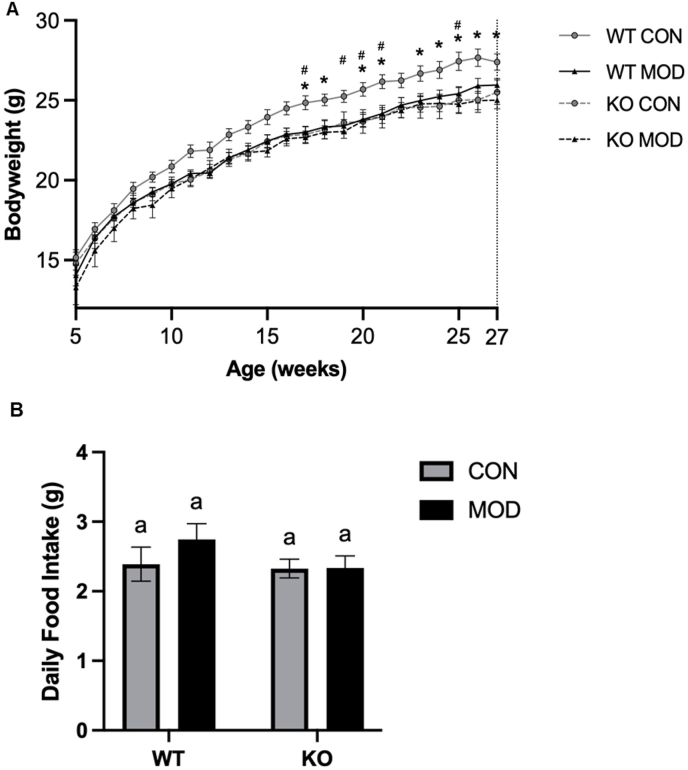
The *hnRNPI* KO mice had altered colonic architecture. (A) Weekly body weight. *n* = 10–11 mice per group. (B) Average daily food intake of WT and KO mice on CON or MOD diet. *n* = 2–4 cages per group. Data are presented as mean ± SEM. ∗Indicates a significant difference between WT-CON and KO-CON, and #indicates a significant difference between WT-CON and WT-MOD *(P*<0.05). Mean with same letter are not significantly different at *P* < 0.05. CON, control protein diet; hnRNPI, heterogeneous nuclear ribonucleoprotein I; KO, knockout; MOD, nutrient-dense modified diet; WT, wild type.

KO mice exhibited lower body weight than WT mice when fed the CON diet, and WT mice fed the MOD diet had lower body weight than the WT mice fed the CON diet. Body weight was monitored from 5 to 27 wk of age. In the CON group, KO mice were significantly lighter than WT mice beginning at ∼17 wk of age (Figure 1A). In contrast, no significant differences in body weight were observed between KO and WT mice in the MOD group. Mean daily food intake was also measured in both dietary groups. No significant differences in food intake were detected among the experimental groups (Figure 1B). These results indicate that the reduced body weight observed in KO mice under the CON diet was not attributable to differences in food consumption. Instead, this phenotype may reflect impaired intestinal function in KO mice, potentially leading to reduced efficiency of digestion and nutrient absorption [8,10].

### The MOD diet restores goblet cell and mucin loss in *hnRNPI* KO mice

**FIGURE 2:**
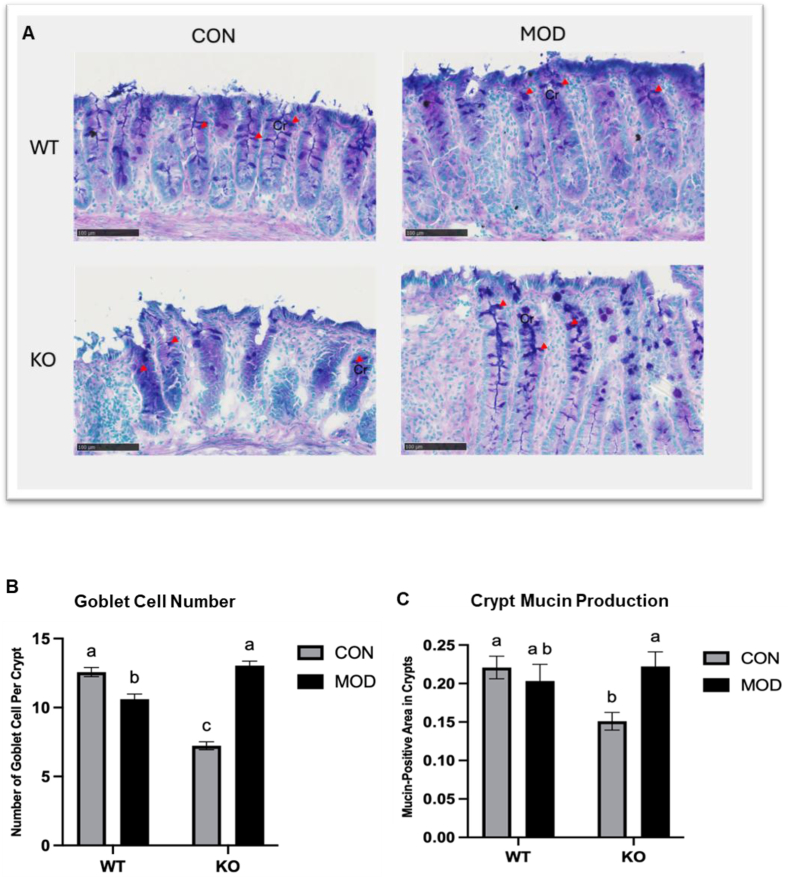
MOD diet restored gut barrier functions in the inflamed colon. (A) AB/PAS-stained distal colon sections. Goblet cells appear as dark purple, indicated by the red arrow. (B) Goblet cells count per crypt. (C) Mucin-positive area in colonic crypts. *n* = 4 mice per group. Data are presented as mean ± SEM. Means with different letters are significantly different at *P* < 0.05, groups sharing the same letter are not significantly different. Scale bars, 100 μm. AB/PAS, Alcian Blue/periodic acid–Schiff; CON, control protein diet; KO, knockout; MOD, nutrient-dense modified diet; WT, wild type.

Goblet cells and mucin production are essential for maintaining intestinal barrier integrity and protecting against inflammation; therefore, we next examined the effect of the MOD diet on colonic goblet cells and mucin content in WT and KO mice. Goblet cells were visualized using AB/PAS staining (Figure 2A), and goblet cell numbers were quantified across genotypes and dietary groups (Figure 2B). Importantly, the MOD diet significantly increased the number of goblet cells in KO mice compared with KO-CON. In contrast, WT-MOD mice showed a modest reduction in goblet cells compared with WT-CON. This alteration of goblet cells in our study is consistent with previous findings [23]. We further quantified mucin content in the crypts by measuring the mucin-positive area (Figure 2C). KO-CON mice showed a marked reduction in mucin compared with WT-CON. Importantly, KO-MOD mice showed a significant restoration of mucin concentrations, reaching values comparable to those of WT groups. Together, these results indicate that a MOD diet may restore gut barrier properties by increasing goblet cell number and mucin production in the inflamed colon of *hnRNPI* KO mice.

### MOD diet alters the CD4^+^ T-cell subpopulations in *hnRNPI* KO mice by decreasing IL-17^+^ IFN-γ^+^ CD4^+^ T cells

**FIGURE 3:**
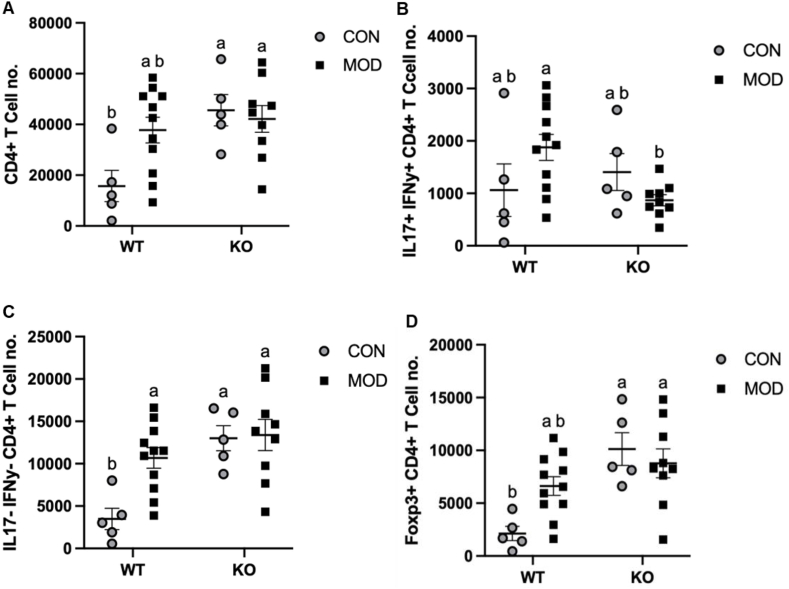
MOD diet altered CD4^+^ T-cell subsets in WT and KO mice. (A) Total CD4^+^ T cells in WT and KO mice on CON or MOD diet, (B) number of IL-17^+^ IFN-γ^+^ CD4^+^ T cells, and (C) number of IL-17^–^ IFN-γ^–^ CD4^+^ T cells in WT and KO mice on CON or MOD diet; (D) Number of Treg (Foxp3^+^ CD4^+^) in WT and KO mice on CON or MOD diet. *n* = 10–11 mice per group. Data are presented as mean ± SEM. Means with different letters are significantly different at *P* value < 0.05, whereas means sharing at least one letter are not significantly different. CON, control protein diet; IFN, interferon; KO, knockout; MOD, nutrient-dense modified diet; Treg, regulatory T-cell; WT, wild type.

We further analyzed immune cell populations in *hnRNPI* KO mice by flow cytometry after dietary treatments. We analyzed the T-cell population using IL-17, IFN-γ, and FOXP3 to identify T-cell subpopulations. Flow cytometry revealed significant differences in the abundance and proportions of CD4^+^ T-cell subtypes. Compared with WT-CON, KO-CON, and KO-MOD mice showed a significant increase in total CD4^+^ T cells (Figure 3A). Interestingly, we observed a significantly lower IL-17^+^ IFN-γ^+^ CD4^+^ T-cell subset in KO-MOD than in WT-MOD (Figure 3B), suggesting that the MOD diet suppresses these T-cell subsets during colon inflammation. In addition, the MOD diet increased the number of IL-17^–^ IFN-γ^–^ CD4^+^ T cells in WT mice, a subset of T cells devoid of IL-17 and IFN-γ expression (Figure 3C). These findings indicate that the MOD diet decreases T helper cells producing IL-17 and IFN-γ and, interestingly, may affect alternative T helper cell lineages that do not produce IL-17 and IFN-γ, which function differently during colon inflammation in *hnRNPI* KO mice. Furthermore, we measured the Treg cell population in the colon of *hnRNPI* KO mice. Interestingly, we found that the Treg cell population, Foxp3^+^ CD4^+^ T cells, was elevated in KO mice. KO-CON mice demonstrated ∼2-fold more Tregs than WT-CON (Figure 3D), indicating an active Treg recruitment in the colon of *hnRNPI* KO mice. This finding indicates the alteration of the immune response in *hnRNPI* KO mice during colon inflammation.

### MOD diet shapes the gut microbiota by altering taxa abundance and enriching *Dubosiella* sp. in KO mice

**FIGURE 4:**
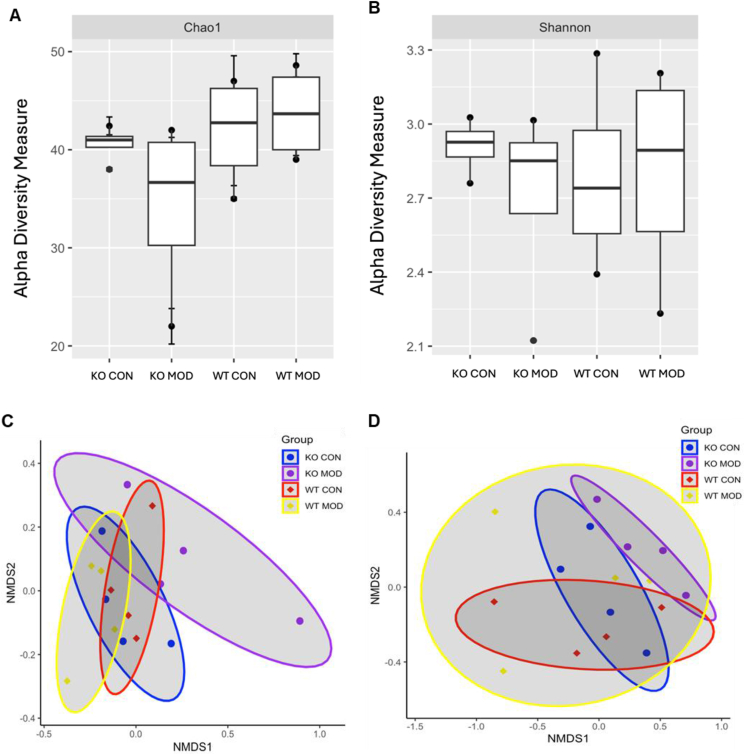
The MOD diet altered gut microbiota beta diversity. (A) Chao1 and (B) Shannon index of WT and KO mice on CON or MOD diet. NMDS ordinations of fecal microbiota using (C) binary Jaccard (presence–absence) and (D) Bray–Curtis (abundance-weighted) dissimilarities of WT and KO mice on CON or MOD diet. *n* = 4 mice per group. CON, control protein diet; KO, knockout; MOD, nutrient-dense modified diet; NMDS, nonmetric multidimensional scaling; WT, wild type.

To understand the change in colon microbial homeostasis by the MOD diet, we investigated the gut microbiota composition in the fecal samples. Alpha diversity (Chao1 and Shannon) did not indicate significant changes between CON and MOD diets (Figure 4A, B). Meanwhile, beta diversity using Jaccard analysis demonstrated significant compositional separation (*P* = 0.019, Figure 4C), whereas the abundance-weighted Bray–Curtis distances did not reach significance (*P* = 0.124, Figure 4D). This pattern suggests that dietary intervention primarily altered the presence of taxa rather than their relative abundance. These results led us to further characterize gut microbiota between CON and MOD diets in WT and KO mice.

**FIGURE 5:**
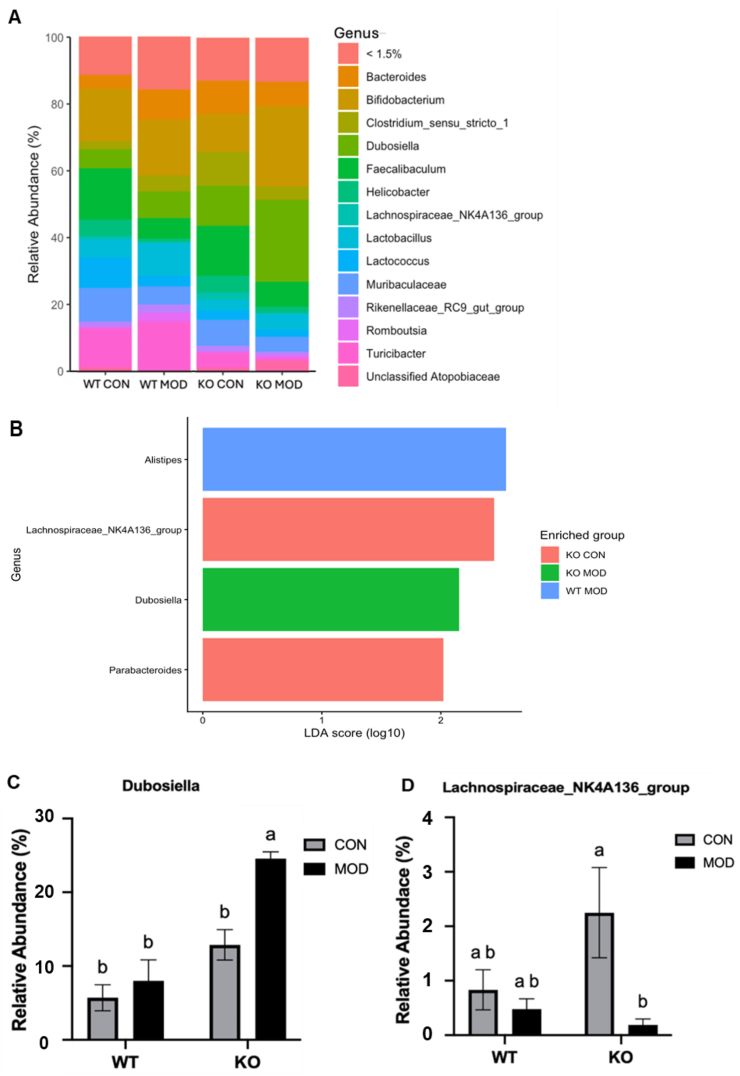
MOD diet caused changes in microbial taxa. (A) Relative abundance of microbial communities at the genus level in WT and KO mice on CON or MOD diet. Genus with relative abundance <1.5% across samples were combined into a single category (<1.5%) for visualization. (B) LEfSe analysis showing bacterial genera differentially enriched among groups. The LDA score estimates the effect size of each differentially abundant feature in each taxon, reflecting the differences in relative abundance between groups, and identifies microbial taxa signatures in each group (LDA >2.0, *P* < 0.05). (C) Relative abundance of *Dubosiella* in WT and KO mice on CON or MOD diet. (D) Relative abundance of *Lachnospiraceae NK4A136 group* in WT and KO mice on CON or MOD diet. *n* = 4 mice per group. Means with different letters are significantly different at *P* value < 0.05, whereas means sharing at least one letter are not significantly different. Data are presented as mean ± SD. CON, control protein diet; KO, knockout; LDA, linear discriminant analysis; LEfSe, linear discriminant analysis effect size; MOD, nutrient-dense modified diet; WT, wild type.

Analysis of 16s-rRNA sequencing using QIIME2 identified a shift of microbes at the genus level between WT and KO mice, as well as between CON and MOD diets (Figure 5A). The MOD diet altered the gut microbial composition, as determined by changes in the relative abundance of bacterial taxa (Figure 5A). Using LEfSe analysis, we identified significant changes in KO mice fed the MOD diet (Figure 5B). Specifically, *Dubosiella* sp. is identified as a gut microbiota marker enriched in KO mice fed the MOD diet (Figure 5C). Meanwhile, Lachnospiraceae NK4A136 group is more abundant in the KO-CON mice, and critically, MOD feeding was effective in reducing the potentially detrimental microbe (Figure 5D). Notably, *Dubosiella* sp. is a potential probiotic that can restore colon homeostasis during inflammation [26], suggesting that the potential function of enriching *Dubosiella* sp. under a MOD diet could modulate immune responses and enhance the number of goblet cells in KO mice. Although LEfSe analysis identified several taxa with differential abundance among groups, we focused our downstream interpretation on *Dubosiella* and the Lachnospiraceae NK4A136 group, as these taxa showed clearer abundance differences and potential biological relevance, whereas the remaining taxa were present at very low relative abundance across samples.

## Discussion

The current study first demonstrated that a MOD could restore gut homeostasis during inflammation. Our data reveal that the MOD restores colonic goblet cell number and mucin content and alters key inflammatory mediators in *hnRNPI* KO mice. Although we did not observe a significant difference in body weight change between the CON and MOD groups, we found that MOD could alter the T-cell population by decreasing the number of IL17^+^ IFN-γ^+^ CD4^+^ T cells in KO mice. More critically, we discovered that MOD alters gut microbiota composition, particularly at the genus level, by increasing the abundance of *Dubosiella* sp. in *hnRNPI* KO mice.

The MOD diet appeared to promote epithelial renewal and differentiation along the crypt-surface axis, suggesting improved mucosal regeneration. In line with this, it was supported by a significant increase in the number of goblet cells in *hnRNPI* KO mice fed MOD diet compared with WT mice, indicating that MOD may facilitate epithelial maturation and mucus-secreting cell differentiation during inflammation. This finding is consistent with previous studies reporting an increase in goblet cells after supplementation with high-protein diets [23,24]. Goblet cells, a specialized type of epithelial cell, are important for producing mucus and protecting the intestinal lining from direct contact with the lumen. During gut inflammation, such as colitis, goblet cell numbers are reduced [13]. As shown in KO-CON mice, goblet cell numbers were significantly decreased compared with WT-CON and KO-MOD, consistent with the previous result [8]. Interestingly, the MOD diet was associated with fewer goblet cells in WT mice compared with a CON diet. Although the mechanisms underlying this observation remain unclear, it may reflect potential adverse effects of higher protein intake in otherwise healthy individuals. Even though excess undigested dietary protein reaches the large intestine, where bacterial fermentation produces metabolites that can negatively affect colonic health and promote intestinal inflammation [27], we speculate that this effect may be influenced by the level of dietary protein intake. However, in our study, we demonstrated that supplementation with optimized protein intake could restore goblet cell numbers and mucin production in KO-MOD mice.

In addition to goblet cell recovery, we also observed a restoration of mucin content within the crypts of KO-MOD mice compared with KO-CON mice. Mucin restoration indicates that a moderate-protein diet can promote mucus layer replenishment in the inflamed colon. This is particularly relevant because the mucus layer is a critical component of the epithelial barrier, providing protection against luminal antigens and bacterial invasion. Previous studies have shown that impaired mucin production is strongly associated with increased intestinal permeability and susceptibility to colitis [28]. Thus, the increase in mucin content observed in KO-MOD mice suggests that moderate-protein intake increases goblet cell numbers and may help restore mucosal barrier impairment during colitis [10].

During gut inflammation, proinflammatory cytokines are produced by immune cells, exacerbating the immune response in the gut [24,29]. In a previous study, Xu et al. [10] observed reduced gene expression Concentrations of proinflammatory cytokines, such as *Il6*, *Il1β*, *Cxcl1*, and *Ccl2* in KO-MOD compared with KO-CON. They also found that MOD supplementation altered the number of CD4^+^ T cells. This prompted us to characterize the subpopulation of T cells, including Th and Treg cells. T helper cell accumulation in the gut promotes the inflammation through the secretion of IL-17 and IFN-γ; on the other hand, Treg cells could ameliorate the gut inflammation and promote gut healing [16,26]. In the present study, KO mice fed the MOD diet had significantly less IL-17^+^ IFN-γ^+^ CD4^+^ T-cell subpopulation compared with WT-MOD, although no significant difference was observed between KO-MOD and KO-CON. This finding suggests that MOD supplementation may dampen the expression of the inflammatory Th cells, including Th17 and Th1. During colitis progression, Th17 cells cause inflammation and promote an excessive immune response, whereas Treg cells help suppress it [3].

The optimization of protein consumption (∼30% kcal) has been associated with improved mucosal healing, via beneficial shifts in gut microbiota or the provision of anti-inflammatory amino acids [21,30]. We further deepened our analysis by investigating fecal gut microbiota composition after MOD supplementation. We observed an increased *Dubosiella* genus in fecal samples of KO mice fed the MOD diet, correcting the significantly diminished *Dubosiella* genus in *hnRNPI* KO mice [9,31]. *Dubosiella* has emerged as a potentially beneficial microbe with probiotic properties [9,32,33]. This is also supported by previous findings that *Dubosiella* was significantly reduced during colon inflammation. Interestingly, *Dubosiella* also showed antiaging properties [31], preventing the colonization of pathogenic bacteria [34]. *Dubosiella newyorkensis*, one of the species in *Dubosiella* genus, is known to produce amino acids, such as L-lysine [26] and tryptophan derivatives, such as n-formyltryptophan and indole-3-carboxaldehyde [9]. In line with these findings, we observed the restoration of colon health in KO mice by MOD, evidenced by increased goblet cell numbers and restored mucin production. We suggest that the MOD diet may promote the abundance of the *Dubosiella* genus and produce beneficial metabolites to restore colon health in *hnRNPI* KO mice.

Our results indicated that the KO-CON mice showed the highest inflammatory phenotype, as indicated by the loss of goblet cells, the loss of body weight, and increased concentration of CD4^+^ T-cell infiltration, which represents the most pronounced inflammatory response; this is in accordance with the enrichment of the Lachnospiraceae NK4A136 group. Feeding the MOD diet to the KO mice resulted in the reduction of both inflammation and the abundance of the Lachnospiraceae NK4A136 group. Lachnospiraceae NK4A136 group may thrive and increase during inflammation due to possible traits like oxygen tolerance, mucin-degrading enzymes, or the metabolism of glycan by the host [[35], [36], [37]]. Some members of the Lachnospiraceae family are known to have anti-inflammatory effects, whereas others are linked to the disruption of the gut barrier, mucosal erosion, and immune stimulation in IBD and colitis mouse models [[38], [39], [40]]. Further investigations are needed to determine whether the Lachnospiraceae NK4A136 group is merely a dysbiosis microbe or a contributing agent to intestinal barrier defects and immune dysregulation.

Nevertheless, several limitations should be considered in interpreting the findings of this study. First, the dietary intervention involved chronic feeding of a single protein source (casein), and therefore, the findings may not necessarily generalize to other protein sources, including plant-based proteins. In addition, age-related changes in immune responses could potentially confound some observations between younger and older mice. Second, improvements in gut barrier function were inferred primarily from histological observations, including restoration of goblet cell numbers and increased mucin production, which are key structural components of the intestinal mucus barrier. However, intestinal permeability was not directly measured in the present study. Future studies should, therefore, include functional assessments of barrier integrity to better characterize the dietary effects on goblet cell function and mucus barrier integrity. Furthermore, single-cell RNA sequencing of MOD diet-fed KO mice will delineate the remodeling of cell lineage derived from stem cell populations, identifying potential mechanisms of goblet cell enrichment by the MOD diet. Certain limitations need to be addressed in future research: findings from the present study reflect chronic feeding of a single protein source (casein) and may not be generalizable to alternative protein sources (e.g., plant-based). In addition, age-related changes in immunity could confound some observations in young and old mice. In addition to the results from the current study on goblet cell numbers and mucin production, future research is needed to functionally characterize the dietary effects on goblet cell function, including, but not limited to, measuring colon permeability. The IL-17^–^ IFN-γ^–^ CD4^+^ T cells subset requires further characterization (e.g., T-cell receptor profiling) to determine whether these cells are naïve, memory, or part of another regulatory subset. Lastly, conducting an *in vitro* study to determine the mechanism by which *Dubosiella* metabolites restore gut health, such as by inducing goblet cell and mucus production, would support the potential use of *Dubosiella* sp., especially *Dubosiella newyorkensis* and its metabolite profile described in the previous study, as future probiotics and postbiotics for preventing gut inflammation.

In summary, an optimized protein diet offsets epithelial damage in *hnRNPI* KO mice, enhances the intestinal barrier by restoring goblet cells and replenishing mucin content, and alters colonic inflammatory responses, accompanied by increased IL-17^–^ IFN-γ^–^ CD4+ T cells and the Treg cell population. Lastly, the optimized protein diet modulates gut microbiota composition by increasing the abundance of *Dubosiella* sp. and decreasing the abundance of the Lachnospiraceae NK4A136 group. Our findings suggest a multifaceted interaction among diet, colon functions, and the gut microbiota. These findings highlight the potential of moderate dietary protein interventions to modulate chronic intestinal inflammation and pave the way for future investigations into protein quality, amino acid composition, and their translational relevance to human IBD management.

## Author contributions

The authors’ responsibilities were as follows – Y-XP, HC: designed the study; JL, LJ, AACP: wrote the manuscript drafts; JL, LJ: conducted experiments and performed statistical analysis; and all authors: read and approved the final manuscript.

## Data availability

Data described in the manuscript, code book, and analytic code will be made publicly and freely available without restriction at (URL).

## Declaration of Generative AI and AI-assisted technologies in the writing process

The author(s) declare that no generative AI or AI-assisted technologies were used in the writing of this manuscript.

## Funding

This project was supported by the USDA Cooperative State Research, Education, and Extension Service (Hatch project numbers # ILLU-698989 and ILLU-698992), the Division of Nutritional Sciences-Vision 20/20, and the Office of the Vice-Chancellor for Research at the University of Illinois Urbana-Champaign.

## Conflict of interest

The authors declare that the research was conducted without any commercial or financial relationships that could potentially create a conflict of interest.
